# Evaluating university emergency resilience with a focus on student safety and health

**DOI:** 10.3389/fpubh.2026.1830542

**Published:** 2026-05-14

**Authors:** Lei Li, Yongqi Guo, Yang Yin

**Affiliations:** School of Civil Engineering, Central South University, Changsha, China

**Keywords:** campus emergency management, fuzzy comprehensive evaluation, indicator system, student safety and health, university emergency resilience

## Abstract

**Background:**

Universities are increasingly exposed to emergencies that threaten student safety and health, yet existing studies on university emergency resilience remain largely institution-centered and pay insufficient attention to student-oriented resilience capacities.

**Methods:**

This study develops a student-oriented university emergency resilience (UER) evaluation framework focused on student safety and health. A five-dimension, 28-indicator system was constructed through semi-structured interviews, thematic analysis and questionnaire survey. Comprehensive weights were determined by integrating the Analytic Hierarchy Process and the coefficient of variation method. Central South University (CSU), China, was selected as the empirical case. Its UER was evaluated using characteristic-function fuzzy comprehensive evaluation, and a priority analysis was further conducted to identify the deficiencies that should be prioritized in remedial action.

**Results:**

CSU achieves a defuzzified comprehensive score of 3.5421, corresponding to Grade 4 (“Good”). Medical and Health Support Capacity and Campus Environment and Facility Safety Readiness perform relatively well, whereas Risk Communication and Information Accessibility is the weakest dimension. The priority analysis identified Academic Flexibility and Adjustment and Psychological Crisis Intervention Services as the most urgent deficiencies.

**Conclusion:**

Although CSU demonstrates a generally good level of UER, its resilience remains uneven across dimensions, with the main weaknesses concentrated in communication, psychosocial support, and post-emergency continuity. This study extends institution-centered UER assessment by introducing a student safety- and health-oriented resilience framework that can support more targeted resilience improvement in universities.

## 1 Introduction

Universities constitute complex socio-organizational systems in which large student populations engage in learning, living, and social activities within relatively concentrated spatial and temporal settings. A wide range of emergency events, such as campus fires, laboratory accidents, extreme weather disruptions, and public health incidents, can directly threaten student safety and health and disrupt the continuity of educational activities. Consequently, the capacity of universities to effectively cope with emergencies has become a critical issue in campus safety governance and institutional risk management (1).

Existing research and practice in university emergency management have predominantly approached emergencies through the lens of emergency management, emphasizing organizational structure (2), information communication (3), emergency plan (4), and so on. While these approaches contribute to improving immediate response efficiency, they tend to focus on isolated stages of emergency handling. In contrast, emergency resilience provides a more integrative perspective by emphasizing the ability of institutions to prepare for potential disruptions, absorb shocks, adapt to evolving conditions, and recover while sustaining essential functions (5). Currently, the concept of emergency resilience has been widely applied in emergency management research (6–8). Some scholars have also evaluated the emergency resilience of different institutions and regions (9–11). However, emergency resilience is often evaluated based on organizational arrangements, physical infrastructure, or resource availability, with limited consideration of how these capacities affect students as the primary risk-exposed group. This institutional focus may obscure important student-level vulnerabilities and experiential factors that shape actual safety and health outcomes during emergencies.

From a student-centered perspective, emergency resilience encompasses not only protection from physical hazards but also the capacity to safeguard health, enhance risk awareness, and ensure access to timely information and support (12). Dimensions such as the availability of medical and psychological services, the effectiveness of risk communication, and students' preparedness to respond to emergency instructions play a critical role in determining safety and health consequences (13). However, these student-oriented dimensions are rarely systematically incorporated into existing frameworks for evaluating university emergency resilience (UER), resulting in an incomplete representation of resilience performance.

In response to these gaps, this study develops a student-oriented framework for evaluating UER, with an explicit focus on student safety and health. By integrating key stages of emergency resilience with student-oriented safety and health dimensions, the proposed framework aims to provide a more comprehensive basis not only for assessing UER, but also for identifying the deficiencies that should be prioritized in subsequent improvement efforts. The findings are therefore expected to offer both theoretical insights into student-oriented UER assessment and practical guidance for strengthening university emergency management through more targeted resilience improvement.

## 2 Methodology

**Figure 1:**
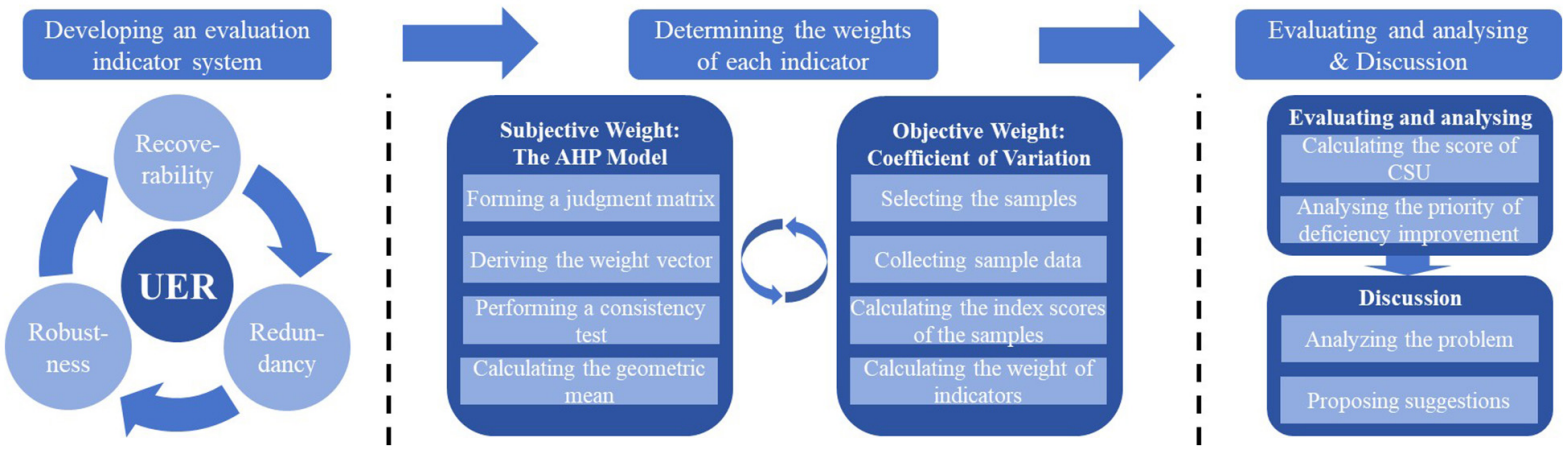
Methodology framework for evaluating UER.

Building on resilience theory and a student safety-and-health perspective, this study develops an indicator-based framework to evaluate UER (Figure 1). First, a resilience-based indicator system is constructed through semi-structured interviews and thematic analysis, producing a structured set of dimensions and indicators with explicit five-level evaluation criteria and subsequent questionnaire-based validation to ensure clarity, relevance, and measurability. Second, indicator weights are quantified by integrating subjective weights derived from the Analytic Hierarchy Process (AHP) with objective weights calculated via the coefficient of variation (CV) methods, so that expert judgment and objective data jointly inform the weighting scheme. Third, based on the indicator scores of Central South University (CSU), the UER level of CSU is evaluated using fuzzy comprehensive evaluation (FCE). Finally, drawing on both the evaluation results and the comprehensive weights, a priority analysis is conducted to identify the deficiencies that should be addressed first, thereby providing a more targeted basis for remedial action and emergency management improvement.

### 2.1 Indicator system development

Resilience theory holds that positive adaptation despite significant adversity is fostered by promotive factors that help people withstand, counteract, or recover from risk exposure (14). In university disaster and emergency research, resilience is commonly framed as an education system's ability to absorb disturbances, self-organize, adapt, and continue delivering core functions before, during, and after shocks (1). Resilience typically encompasses three dimensions: robustness, recoverability, and redundancy. In university emergency management, robustness is the ability to withstand shocks and keep core functions running; recoverability is the ability to restore functions after an incident; and redundancy is the availability of backups and alternatives when primary resources or systems fail. Implementing university emergency management grounded in resilience theory helps schools to make rapid decisions, mobilize resources flexibly, deliver essential support, and conduct robust post-incident reviews, thereby minimizing students' physical and psychological harms while maintaining critical functions and continuously strengthening the campus's capacity to cope with future emergencies.

Based on the above research, evaluating UER is necessary because it translates the abstract notion into an assessable set of capabilities that determine whether a campus can minimize students' physical and psychological harms while maintaining critical functions during sudden events. In this study, semi-structured interviews were conducted with 13 participants to collect concepts and information relevant to university emergency management from a resilience perspective, so as to support preliminary concept elicitation and exploratory indicator development. The interview transcripts were then analyzed using thematic analysis to systematically identify recurring concepts, organize them into coherent dimensions and indicators, and ultimately develop a resilience-based indicator system for evaluating UER.

#### 2.1.1 Semi-structured interviews

This study employed semi-structured interviews to collect concepts and information relevant to university emergency management from a resilience perspective, with the aim of supporting preliminary concept elicitation and exploratory indicator development. Based on this purpose, the sampling principle was to obtain sufficiently rich and conceptually relevant information within a limited sample by covering the major role categories associated with emergency prevention, response, and recovery. Accordingly, because university emergency management involves multiple roles closely related to emergency prevention, response, and recovery, this study classified these roles and established a list of eligible individuals within each category. One participant was selected from each category by simple random selection after confirming willingness to participate. The details of the interviewees were listed in Table 1. This approach ensures that the sample covers different roles across the entire process of emergency management, and provides sufficient conceptual breadth for indicator development.

**Table 1:** Profiles of interviewees.

No.	Role	Relevant experience
1	Director, campus security office	12 years
2	Deputy director, student affairs office	9 years
3	Head, university health service center	15 years
4	Supervisor, psychological counseling center	10 years
5	Manager, facilities & fire safety	8 years
6	Emergency communication	7 years
7	Undergraduate student (senior), student leader	4 years
8	Undergraduate student (junior), resident assistant	3 years
9	Undergraduate student (sophomore), participated in multiple drills	2 years
10	Undergraduate student (freshman), newly on boarded safety training	1 year
11	Master's student, lab safety representative	2 years
12	Master's student, international student representative	2 years
13	PhD student, student affairs liaison	4 years

The interview protocol was designed to guide discussions while allowing flexibility to probe emerging themes. Core questions focused on how university emergencies are managed to protect students' physical and psychological health, including coordination arrangements, resource mobilization, redundancy for critical functions, risk communication, post-incident learning mechanisms and so on. Participants were invited to respond to five main questions regarding UER evaluation:

(1) How does your university organize and execute emergency management with an explicit focus on students' safety and health?
(2) In an emergency, what are the main strengths and gaps in evacuation, safety facilities, security staffing, and continuity of essential services on campus?
(3) What capacities ensure timely medical and psychological support for students during and after emergencies?
(4) How does the university communicate emergency information to students to ensure speed, clarity, and trust?
(5) After an incident, how does the university support students' recovery and return to normal academic and daily life?

Ethical procedures were implemented by obtaining informed consent, assuring anonymity, and securing permission for audio recording; interviews were conducted, recorded, and transcribed verbatim, and transcripts were returned to interviewees for confirmation to enhance content validity.

#### 2.1.2 Thematic analysis

The interview transcripts were analyzed using thematic analysis to systematically identify, interpret, and synthesize recurring themes relevant to UER. After transcription, all texts were read repeatedly to ensure familiarity, followed by line-by-line open coding to generate an initial codebook capturing discrete resilience-related concepts; codes were then iteratively reviewed, refined, merged, or removed, and subsequently aggregated into higher-order themes that represent core resilience capacities and can be operationalized as candidate evaluation indicators. The coding and theme development proceeded until data saturation, and to enhance reliability, only themes reported by at least three participants were retained; finally, the resulting indicators were triangulated with existing resilience and university emergency management literature to strengthen the validity and interpretability of the indicator system. The thematic analysis yielded a resilience-based indicator system comprising five dimensions, including Emergency Governance and Operational Effectiveness, Campus Environment and Facility Safety Readiness, Medical and Health Support Capacity, Risk Communication and Information Accessibility, Post-Emergency Student Support, and 28 indicators. Each dimension is detailed separately in Table 2.

**Table 2:** Thematic analysis of indicators.

Criteria layer	Indicator layer	Description	Resilience attribute	Summary of interview notes
Emergency governance and operational effectiveness (A)	Student-oriented emergency response procedures (A1)	Whether clear, documented, and operational emergency response procedures for students have been established	Robustness	Interviewee 1: “We have a written student-facing procedure, and we revise it after drills so the steps are clearer while emergency response”
				Interviewee 8: “What works is a short, fixed process students can follow; when the guidance is only ‘general principles,' it's hard to act in the moment”
	Emergency communication channels (A2)	Number of official emergency communication channels accessible to students	Redundancy	Interviewee 10: “If I only get notice from one place, I might miss it; when alerts come through several official channels, I'm more likely to see and follow them”
	Integration of student safety and health provisions (A3)	Degree to which student safety and health considerations are embedded in emergency management regulations	Robustness	Interviewee 2: “Student safety and health are not just slogans—we need them written into the rules as concrete requirements, like medical support, psychological support, and basic living arrangements”
	Student participation in emergency training and drills (A4)	Frequency and coverage of student participation in emergency training and drills	Robustness	Interviewee 7: “When drills happen regularly and cover most students, people know what to do; without routine participation, many students freeze or follow others”
	Post-incident review and improvement mechanism (A5)	Implementation of post-incident reviews and corrective actions following emergencies	Recoverability	Interviewee 2: “After an incident, we should produce a written review, assign corrective actions to specific offices, and track whether they are completed, to prevent similar problems from recurring”
				Interviewee 5: “If the review leads to real fixes, like updating signage, equipment checks, and response process optimizing, and we confirm completion, the next response is noticeably smoother”
	Decision documentation and traceability (A6)	Whether emergency decision-making processes are fully documented and traceable	Recoverability	Interviewee 6: “Key decisions should be logged with time, sender, content, and channel in one system; then we can trace what was decided and what students actually received”
	Establishment and clarity of emergency management authority (A7)	Whether a dedicated emergency management unit is established and whether its responsibilities and authority are clearly defined	Robustness	Interviewee 2: “Clear responsibility boundaries matter. students and departments need to know who is in charge, otherwise instructions become inconsistent and execution slows down”
Campus environment and facility safety readiness (B)	Comprehensive evacuation route assurance (B1)	Whether evacuation routes are clearly designated and accessible under emergency conditions	Robustness	Interviewee 8: “The emergency exits in the teaching buildings and dormitories are usually unobstructed. However, occasionally, goods or bicycles may be piled up in the exits, making them unsuitable for passage”
	Campus safety facilities (B2)	Coverage of alarm systems, firefighting equipment, and emergency lighting	Robustness	Interviewee 5: “We inspect alarms, extinguishers, hydrants, and emergency lights on a schedule; the gaps usually appear in older buildings where devices are present but not fully functional”
	On-campus security personnel allocation (B3)	Adequacy of campus security personnel allocation for student safety	Robustness	Interviewee 1: “From my dispatch experience, if guard posts are thin at night or during peak entry times, it takes longer to control the scene and guide students, especially around dorms and main gates”
	Continuity of essential public services (B4)	Ability to maintain or restore essential services during emergencies	Recoverability	Interviewee 10: “During a short power cut in the dorm, the biggest problem was not knowing how long it would last; without lighting and charging, students got anxious”
				Interviewee 5: “In my work, we treat water and electricity continuity as a safety issue. Once an outage extends, dorm access control, lighting, and basic living support are all affected”
	Redundancy of safety facilities (B5)	Extent to which critical safety facilities are equipped with backup systems	Redundancy	Interviewee 1: “If a key system fails and there's no backup plan, we can only wait for repairs; with redundancy, we can keep basic safety running in the meantime”
Medical and health support capacity (C)	Campus hospital capacity (C1)	Adequacy of campus medical facilities to serve student emergencies	Robustness	Interviewee 3: “When several students come in at the same time, we quickly run out of beds and observation space, so some cases can only be assessed briefly and asked to wait”
				Interviewee 12: “For common problems the campus clinic is fine, but when we had a more serious issue, we felt the campus clinic couldn't handle it well, so we had to go to an outside hospital”
	Medical response time (C2)	Timeliness of medical personnel arrival at emergency sites	Robustness	Interviewee 3: “In emergencies, the first few minutes matter most, if our staff can reach the scene fast and start basic treatment, the situation stays under control”
	Emergency medical equipment coverage (C3)	Coverage of AEDs and first-aid equipment	Robustness	Interviewee 5: “We do have AEDs and first-aid kits, but the problem is access, if they're locked in an office or placed too far away, they won't help in the first min”
				Interviewee 7: “I noticed that the school has recently installed many AED. I think this is very good. In case a student has an emergency, these devices can be put to use promptly”
	Psychological crisis intervention services (C4)	Availability of psychological counseling for emergencies	Recoverability	Interviewee 4: “Students are more willing to seek help when the school tells them clearly ‘where to go and how to book'; if the process is complicated, many just keep it to themselves”
	Support for students with special health needs (C5)	Support mechanisms for students with special health needs	Recoverability	Interviewee 2: “Students with chronic illness, disability, or high mental-health risk need a clear support plan, including who checks on them, how medication or special accommodation is handled, otherwise we scramble during an incident”
				Interviewee 12: “For international students, language and system barriers are real; in an emergency, not knowing what to say or where to go can delay care and make them feel isolated”
	Cooperation with high-level hospitals (C6)	Formal cooperation with tertiary hospitals	Redundancy	Interviewee 3: “When the patients exceed the treatment scope of the campus clinic, the key lies in achieving a smooth referral process. Timely transporting patients to the appropriate specialized hospital and clearly communicating the patient's condition are crucial for saving the patients”
Risk communication and information accessibility (D)	Timeliness of emergency information release (D1)	Speed of official emergency information release	Robustness	Interviewee 8: “When there's no official update for a while, students start guessing in group chats; a quick notice helps people calm down and follow instructions”
	Actionability of information (D2)	Clarity of behavioral guidance in released information	Robustness	Interviewee 2: “A warning isn't enough—students need clear actions, like whether to evacuate, avoid an area, or stay indoors, and who to contact for help”
				Interviewee 10: “The messages I actually follow are the ones that tell me exactly what to do; vague wording makes people ignore it or interpret it differently”
	Multi-channel information dissemination (D3)	Use of multiple channels for information dissemination	Redundancy	Interviewee 6: “We use multiple channels because students' habits differ. SMS for speed, the app for details, and official social media for reach; one channel can't cover everyone”
	Information error-correction mechanism (D4)	Ability to correct misinformation	Recoverability	Interviewee 6: “When wrong information spreads, we correct it through official accounts and pinned messages; the correction has to be fast and clear, not hidden in a long notice”
				Interviewee 13: “Students lose trust quickly when messages conflict; if all channels repeat the same points, it's much easier for student reps to communicate”
	Consistency of authoritative information (D5)	Consistency across official information channels	Robustness	Interviewee 1: “The worst case is different offices sending different instructions; we need one source of truth so students don't get mixed signals”
	Student emergency liaison roles (D6)	Designation of student emergency liaison personnel	Redundancy	Interviewee 2: “Some students need extra support to receive information—like those with disabilities or those off campus—so we plan alternative ways to reach them”
Post-emergency student support (E)	Academic flexibility and adjustment (E1)	Speed of academic recovery after emergencies	Recoverability	Interviewee 2: “After an incident, students mainly ask ‘what about classes'; clear decisions on suspension, online shift, and makeup plans reduce anxiety and keep things from becoming chaotic”
				Interviewee 7: “What helped most was getting a simple update on whether classes were canceled and how assignments would be handled; uncertainty makes students stressed and distracted”
	Coverage of health follow-up services (E2)	Provision of post-emergency health follow-up	Recoverability	Interviewee 8: “After the event, a few students in my dorm kept feeling nervous; it would help if the school checked in and told us where to get support”
	Provision of basic living conditions (E3)	Support for accommodation and meals	Recoverability	Interviewee 5: “If dorm access or dining is disrupted, students' stress rises quickly; temporary arrangements for rooms and meals are a practical part of recovery”
	Financial assistance and fee reductions (E4)	Financial support for affected students	Recoverability	Interviewee 12: “If something happens and you suddenly need to pay for transport or treatment, it's stressful; knowing there is a simple way to apply for support would help a lot”

#### 2.1.3 Indicators testing

Based on the thematic analysis results, this study developed the preliminary UER indicator system, grounded in the lived experiences and practice-oriented judgments of university administrators and enrolled students.

To strengthen methodological rigor, the preliminary dimensions and indicators derived from thematic analysis were systematically cross-checked with established literature on resilience theory and university emergency management, as summarized in Table 3. Specifically, each dimension and indicator was compared with related constructs and capability domains identified in prior studies to examine conceptual consistency. When similar concepts appeared under different labels, their definitions were reviewed and refined to clarify construct boundaries and reduce overlap. Through this step, the preliminary interview-based indicator system was further refined and theoretically anchored before subsequent questionnaire testing.

**Table 3:** Indicator system for UER evaluation.

Criteria layer	Indicator layer		Five-level evaluation criteria	Reference	μ	σ	CV
Emergency governance and operational effectiveness (A)	Student-oriented emergency response procedures (A1)	1	No procedures	(7)	8.31	0.630	0.076
		2	Draft only				
		3	Approved formal document				
		4	Regularly updated				
		5	Regularly updated, drilled, and optimized				
	Emergency communication channels (A2)	1	None	(25)	7.08	0.954	0.135
		2	One channel				
		3	Two channels				
		4	≥3 channels				
		5	≥3 channels covering all communication needs				
	Integration of student safety and health provisions (A3)	1	Not included;	(8)	7.23	0.725	0.100
		2	Mentioned in principle				
		3	Independent clauses				
		4	Systematically integrated				
		5	Annually evaluated and updated				
	Student participation in emergency training and drills (A4)	1	None	(4, 26)	7.62	1.193	0.157
		2	≤ 1 time/year				
		3	Twice/year				
		4	Three times/year				
		5	≥3 times/year with assessment				
	Post-incident review and improvement mechanism (A5)	1	None	(1)	6.85	1.068	0.156
		2	Informal review				
		3	Documented review				
		4	Corrective actions implemented				
		5	≥80% of corrective actions completed				
	Decision documentation and traceability (A6)	1	None	(1)	6.77	1.013	0.150
		2	Incomplete records				
		3	Complete records				
		4	Cross-department traceability				
		5	Digitalized and auditable records				
	Establishment and clarity of emergency management authority (A7)	1	Not established	(27)	7.77	1.013	0.130
		2	No dedicated unit				
		3	Established but unclear authority				
		4	Established with relatively clear authority				
		5	Established with clearly defined authority				
Campus environment and facility safety readiness (B)	Comprehensive evacuation route assurance (B1)	1	No evacuation routes designated	(6)	8.08	0.760	0.094
		2	Draft routes exist but are incomplete or obstructed				
		3	Approved evacuation routes meeting basic accessibility requirements				
		4	Evacuation routes regularly inspected and clearly marked				
		5	Evacuation routes inspected, drilled, and continuously optimized				
	Campus safety facilities (B2)	1	Safety facilities largely absent	(28)	7.15	1.068	0.149
		2	Partial installation with limited coverage				
		3	Facilities formally installed meeting minimum standards				
		4	Facilities regularly inspected and well maintained				
		5	Facilities fully covered, redundantly configured, and optimized				
	On-campus security personnel allocation (B3)	1	No dedicated campus security arrangement	(29)	6.54	0.776	0.119
		2	Limited personnel with unclear responsibilities				
		3	Formal security staffing established				
		4	Security staffing adequate and well coordinated				
		5	Security staffing sufficient, professionally trained, and optimized				
	Continuity of essential public services (B4)	1	No continuity or recovery arrangements	(30)	7.00	1.000	0.143
		2	Informal or *ad hoc* restoration measures				
		3	Formal continuity plans established				
		4	Continuity plans regularly tested and supported by backups				
		5	Essential services rapidly recoverable and optimized				
	Redundancy of safety facilities (B5)	1	No backup systems for safety facilities	(30)	7.46	0.776	0.104
		2	Backup systems exist for a small number of facilities				
		3	Backup systems formally installed for key facilities				
		4	Backup systems systematically deployed and tested				
		5	Backup systems comprehensive and optimized				
Medical and health support capacity (C)	Campus hospital capacity (C1)	1	No campus medical facilities	(31)	7.38	1.261	0.171
		2	Very limited medical capacity				
		3	Formal medical facilities meeting basic standards				
		4	Facilities adequately equipped and assessed				
		5	Facilities robust, scalable, and optimized				
	Medical response time (C2)	1	No defined medical response mechanism	(32)	8.23	0.725	0.088
		2	Medical response slow and uncoordinated				
		3	Formal procedures ensure timely arrival				
		4	Response rapid and routinely evaluated				
		5	Response highly efficient and optimized				
	Emergency medical equipment coverage (C3)	1	Equipment largely unavailable	(33)	8.23	0.725	0.088
		2	Limited or uneven equipment coverage				
		3	Equipment meets minimum coverage standards				
		4	Coverage extensive and regularly inspected				
		5	Coverage comprehensive and optimized				
	Psychological crisis intervention services (C4)	1	No psychological crisis services	(34)	7.54	0.967	0.128
		2	Informal or temporary support				
		3	Formal counseling services established				
		4	Services adequately staffed and routine				
		5	Services comprehensive and continuously improved				
	Support for students with special health needs (C5)	1	No targeted support mechanisms	(35)	6.62	0.650	0.098
		2	Limited and *ad hoc* support				
		3	Formal support mechanisms documented				
		4	Support systematically delivered and monitored				
		5	Support comprehensive and optimized				
	Cooperation with high-level hospitals (C6)	1	No cooperation with external hospitals	(36)	7.38	0.870	0.118
		2	Informal or case-by-case cooperation				
		3	Formal cooperation agreement established				
		4	Multiple agreements with coordinated procedures				
		5	Standardized referral, drills, and optimization				
Risk communication and information accessibility (D)	Timeliness of emergency information release (D1)	1	No formal information release mechanism	(3)	7.00	1.080	0.154
		2	Delayed and inconsistent release				
		3	Formal procedures ensure timely release				
		4	Rapid release with monitoring				
		5	Real-time, coordinated, optimized release				
	Actionability of information (D2)	1	No actionable guidance	(37)	7.62	0.961	0.126
		2	Limited behavioral guidance				
		3	Clear actionable guidance provided				
		4	Guidance systematic and user-oriented				
		5	Guidance adaptive and optimized				
	Multi-channel information dissemination (D3)	1	Single information channel	(3)	7.54	0.776	0.103
		2	Two channels used				
		3	Three channels used				
		4	Four channels used				
		5	Five or more coordinated channels				
	Information error-correction mechanism (D4)	1	No error-correction mechanism	(38)	6.31	0.480	0.076
		2	Correction slow and *ad hoc*				
		3	Formal correction procedures exist				
		4	Correction timely and monitored				
		5	Correction rapid and optimized				
	Consistency of authoritative information (D5)	1	Information inconsistent	(22, 39)	6.69	1.109	0.166
		2	Partially inconsistent				
		3	Generally consistent				
		4	Minor discrepancies				
		5	Fully consistent				
	Student emergency liaison roles (D6)	1	No student liaison roles	(40)	6.38	0.650	0.102
		2	Limited informal liaison roles				
		3	Formal liaison roles established				
		4	Roles widely implemented and coordinated				
		5	Roles institutionalized and optimized				
Post-emergency student support (E)	Academic flexibility and adjustment (E1)	1	No academic adjustment measures	(8, 41)	8.15	0.689	0.085
		2	*Ad hoc* academic adjustments				
		3	Formal academic adjustment policies				
		4	Adjustments systematically implemented				
		5	Adjustments rapid and optimized				
	Coverage of health follow-up services (E2)	1	No health follow-up services	(42)	6.92	0.760	0.110
		2	Limited follow-up coverage				
		3	Partial coverage implemented				
		4	Majority of students covered				
		5	Full coverage ensured				
	Provision of basic living conditions (E3)	1	No living support provided	(43)	7.69	0.751	0.098
		2	Partial living support				
		3	Basic living support provided				
		4	Widespread support provided				
		5	Comprehensive support ensured				
	Financial assistance and fee reductions (E4)	1	No financial assistance provided	(44)	7.15	0.899	0.126
		2	Limited financial assistance				
		3	Formal financial assistance mechanisms				
		4	Assistance covers majority of students				
		5	Comprehensive financial support				

The resulting framework translates UER into an assessable set of dimensions and indicators that reflect a university's capacity to minimize students' physical and psychological harms while maintaining critical functions during sudden events.

For the purpose of consistent evaluation, the performance of each resilience indicator is classified into five levels, where Level 5 represents the strongest resilience capacity and Level 1 represents the weakest. These grading standards were established by synthesizing relevant regulations and guidelines, published research on resilience and UER, and suggestions from the study participants. For indicators that are primarily qualitative, level judgments were determined through respondents' recommendations with reference to institutional documents such as emergency plans, drill records, training materials, and after-action reports, ensuring that each level corresponds to observable evidence rather than subjective impressions.

To validate the proposed UER indicator system, a questionnaire survey was administered to the same 13 respondents listed in the interviewee profile table in order to assess whether the indicators were perceived as important and conceptually appropriate for evaluating UER. Each respondent was asked to rate the importance of every indicator using a 9-point Likert scale (1 = least important; 9 = most important) and provide open-ended suggestions to add, modify, or delete indicators that were unclear, redundant, or difficult to operationalize. Following the statistical validation approach, three parameters for each indicator were calculated: mean score (μ), standard deviation (σ), and *CV*. Higher μ indicates higher perceived importance, and lower σ/*CV* indicates stronger agreement across respondents; indicators with high μ and low *CV* were retained, whereas those with relatively low μ or high *CV* were revised or removed. The mean scores (μ) of all indicators were above 6, and all *CV* values were below 0.2. Results indicate that experts consistently agree that these indicators are important, and the developed indicator system can be used for evaluating UER.

### 2.2 Weighting calculation

Indicator weights were determined through an integrated procedure combining subjective expert judgment and objective data dispersion, followed by the synthesis of comprehensive weights for subsequent evaluation, as commonly adopted in multi-criteria evaluating studies.

First, subjective weights were derived using a group AHP, a widely adopted multi-criteria decision-making method for addressing complex evaluation problems involving multiple interrelated criteria and uncertainty. Thirteen experts with experience in university emergency management, student safety governance, and health support conducted pairwise comparisons of indicators. Individual judgments were aggregated using the geometric mean to form a group judgment matrix (As shown in Equation 1), which is a standard aggregation approach in group AHP applications (15). Indicator weights were derived using the eigenvector method and normalized to obtain the subjective weight vector.


$$\begin{array}{l} A=\left[a_{ij}\right] \end{array}$$ (1)

Where *a*_*ij*_ represents the relative importance of indicator *i* over indicator *j*, satisfying *a*_*ij*_ = 1/*a*_*ji*_ and *a*_*ii*_ = 1. Based on the judgment matrix, indicator weights were obtained using the eigenvector method and normalized as shown in Equation 2.


$$\begin{array}{l} W^{sub}=\left(w_{1}^{sub},w_{2}^{sub},...,w_{m}^{sub}\right),\overset{m}{\underset{j=1}{\sum }}w_{j}^{sub}=1 \end{array}$$ (2)

The logical consistency of expert judgments was examined using the consistency ratio (*CR*), with *CR* < 0.10 indicating acceptable consistency, as commonly applied in AHP-based studies. For multi-level indicator systems, local subjective weights were further synthesized to obtain global subjective weights.

Second, objective weights were calculated based on the *CV* to reflect the dispersion of indicator scores across the sampled universities, which has been widely used to capture objective information contained in indicator variability (16). Let *CV*_*j*_ denote the CV of indicator *j*; objective weights were obtained as shown in Equations 3 and 4.


$$\begin{array}{l} CV_{j}=\frac{s_{j}}{\bar{x_{j}}} \end{array}$$ (3)

$$\begin{array}{l} \bar{x_{j}}=\frac{1}{n}\overset{n}{\underset{i=1}{\sum }}x_{ij}\;,\;s_{j}=\sqrt{\frac{1}{n-1}\overset{n}{\underset{i=1}{\sum }}{\left(x_{ij}-\bar{x_{j}}\right)}^{2}} \end{array}$$ (4)

As shown in Equation 5, objective weights are obtained by normalizing the *CV* values across all indicators.


$$\begin{array}{l} {w_{j}}^{obj}=\frac{CV_{j}}{\sum_{k=1}^{m}CV_{k}}\;,\;\overset{m}{\underset{j=1}{\sum }}{w_{j}}^{obj}=1 \end{array}$$ (5)

Indicators exhibiting greater variability among universities were thus assigned higher objective weights.

Finally, as shown in Equation 6, subjective and objective weights were integrated to form comprehensive indicator weights through linear combination, which has been commonly adopted to balance expert judgment and data-driven information in evaluation studies.


$$\begin{array}{l} W_{j}=\alpha {W_{j}}^{sub}+\left(1-\alpha \right){W_{j}}^{obj} \end{array}$$ (6)

Where α represents the relative contribution of subjective and objective information. Following related studies, α = 0.5 was adopted to assign equal importance to subjective and objective components (17). The resulting comprehensive weights were used not only as the weighting input for subsequent aggregation and evaluation, but also as an indicator of the relative importance of observed deficiencies in the priority analysis. In this sense, the weighting scheme serves both evaluative and remedial purposes.

### 2.3 Evaluation calculation

To implement FCE under a deterministic grading framework, indicator grades are converted into a membership-based representation. Each indicator is assessed according to clearly defined five-level evaluation criteria, and the assigned grades are derived from institutional documents and objective data. Accordingly, a 0–1 characteristic membership function is adopted, whereby each indicator fully belongs to its corresponding grade (18). Based on this specification, a membership matrix is constructed and aggregated using a weighted synthesis operator to obtain the comprehensive membership distribution across evaluation grades. This approach preserves the determinacy of rule-based grading while forming an overall grade structure through multi-indicator weighted aggregation. Therefore, the fuzziness in this study is primarily reflected at the aggregation level in the distribution across grades rather than in continuous membership modeling at the individual indicator level.

Let *r*_*ij*_ denote the membership degree of indicator *i* (*i* = 1, …, *n*) to evaluation grade *v*_*j*_ (*j* = 1, …, *m*; *m* = 5). For a deterministic indicator grade *x*_*i*_, the membership mapping is defined as shown in Equation 7.


$$\begin{array}{l} r_{ij}=\{\begin{array}{c} 1,\;x_{i}\;={\;v}_{j}\\ 0,\;{\;x}_{i}\;\neq {\;v}_{j} \end{array} \end{array}$$ (7)

Accordingly, as shown in Equation 8, the grades are mapped into a membership matrix $R=\left[r_{ij}\right]\in R^{n\times m}$, where each row contains a single non-zero element corresponding to the assigned grade. *W* = (*w*_1_, ..., *w*_*n*_) this study applies a weighted synthesis operator to aggregate contributions across indicators. Compared with max–min (Zadeh-type) operators that may over-emphasize extreme memberships and lead to information loss, weighted synthesis retains the contribution of each indicator and has been widely used in recent FCE-based evaluating studies (19).


$$\begin{array}{l} B=W⊗R,\;b_{j}=\overset{n}{\underset{i=1}{\sum }}\left(w_{i}\times r_{ij}\right) \end{array}$$ (8)

Where *B* = (*b*_1_, ..., *b*_*m*_) represents the aggregated membership distribution over the evaluation grades and satisfies $\overset{m}{\underset{j=1}{\sum }}b_{j}=\;1$.

To transform the fuzzy membership distribution into a scalar resilience index, the grade expectation method (as shown in Equation 9) is applied.


$$\begin{array}{l} S=\overset{m}{\underset{j=1}{\sum }}b_{j}v_{j} \end{array}$$ (9)

Where *v*_*j*_ denotes the numerical value assigned to grade *j*.

The evaluation grade set is defined as shown in Equation 10.


$$\begin{array}{l} V=\left\{v_{1},v_{2},v_{3},v_{4},v_{5}\right\} \end{array}$$ (10)

To operationalize the evaluation scale, five ordered grades were defined to represent progressively increasing levels of emergency resilience performance. The definitions of each grade are presented in Table 4.

**Table 4:** Definition of the five-grade evaluation scale.

Grade	Level	Description
*v*1	Very poor	Absence of systematic mechanisms or institutional arrangements
*v*2	Poor	Initial or fragmented arrangements with limited operational effectiveness
*v*3	Average	Formally established mechanisms meeting basic functional requirements
*v*4	Good	Well-developed and operationalized systems with regular implementation
*v*5	Excellent	Institutionalized, optimized, and continuously improved resilience capacity

These levels are conceptually aligned with the five-level evaluation criteria defined for individual indicators and reflect a progression from structural absence to institutional maturity. This formulation ensures that the overall score captures both the weighted contribution of indicators and the ordinal structure of resilience development stages.

### 2.4 Priority calculation

Based on the previous assessment and calculation results, in order to clearly identify the existing deficiencies and determine which deficiencies should be addressed first in emergency management practices, this study uses priority analysis to convert CSU's identified weaknesses into an actionable sequence of improvements. Specifically, as shown in Equation 11, for each indicator, a deficiency gap was defined as the distance between the observed grade and the optimal grade.


$$\begin{array}{l} Gap_{i}=5-G_{i} \end{array}$$ (11)

Where *G*_*i*_denotes the observed grade of indicator *i*. A priority score was then calculated by combining the deficiency gap with the comprehensive weight of each indicator (As shown in Equation 12).


$$\begin{array}{l} Priority_{i}=W_{i}\times Gap_{i} \end{array}$$ (12)

Where *W*_*i*_ is the comprehensive weight. Under this formulation, a higher priority score indicates that the indicator is not only underperforming, but also more important to overall UER, and therefore should receive greater managerial attention.

### 2.5 Case selection

To examine the applicability of the proposed UER evaluation framework in a real university context, a case study was conducted. Central South University (CSU), located in Changsha, Hunan Province, China, was selected as the empirical case for applying the proposed UER evaluation framework. CSU was chosen for three main reasons. First, its emergency management practices and student support systems are broadly representative of large Chinese higher education institutions, making it a suitable case for examining the applicability of the proposed framework. Second, the university maintains relatively comprehensive institutional documentation and administrative records, which provide a reliable basis for indicator assessment. Third, as a comprehensive university with diverse academic disciplines and a large student population, CSU offers a relevant setting for examining university emergency resilience from a student safety-and-health perspective.

Case data were collected from official university documents, administrative records, and questionnaire materials, and were subsequently organized according to the developed indicator system. For qualitative indicators, grades were assigned with reference to the predefined five-level evaluation criteria and supporting institutional documents. For quantitative indicators, grades were determined based on measurable data provided by the university and then matched to the corresponding evaluation criteria.

## 3 Results

### 3.1 Case data

The CSU case data, organized according to the developed UER indicator system, are presented in Table 5. These data serve as the basis for the subsequent weighting results, evaluation results, and priority results.

**Table 5:** CSU case study data: indicators and evaluation results.

Indicator	Grades	Evaluation criteria
Student-oriented emergency response procedures (A1)	4	A clear, written emergency response process is in place, with continuous improvement
Emergency communication channels (A2)	4	Multiple emergency communication channels are available, though a dedicated emergency notification system is not in place
Integration of student safety and health provisions (A3)	3	Student safety is integrated into the emergency plan, but health provisions are not yet fully separate
Student participation in emergency training and drills (A4)	4	Regular emergency training and drills are conducted, with more than 3 sessions per year, but no formal assessment mechanism is in place
Post-incident review and improvement mechanism (A5)	4	Post-emergency reviews are required, with rectification measures, though no specific evaluation of the corrections is conducted
Decision documentation and traceability (A6)	3	Emergency decisions are documented, but cross-department tracking records are not maintained
Establishment and clarity of emergency management authority (A7)	4	Multiple emergency management departments are established, but no central emergency management office exists
Comprehensive evacuation route assurance (B1)	4	The school ensures evacuation routes are clear and conducts regular fire drills to guarantee high evacuation capacity
Campus safety facilities (B2)	4	Safety facilities are well-maintained, with regular inspections and proper functioning
On-campus security personnel allocation (B3)	5	The ratio of security personnel to students is 1:320, meeting high security requirements
Continuity of essential public services (B4)	4	Basic services, such as water and electricity, are quickly restored during emergencies
Redundancy of safety facilities (B5)	3	Key safety facilities, such as fire extinguishers, have backup measures, though not all facilities are covered
Campus hospital capacity (C1)	5	The university hospital has 8.3 beds per 10,000 students, meeting emergency treatment needs
Medical response time (C2)	4	The attached university hospital ensures timely medical response during emergencies
Emergency medical equipment coverage (C3)	4	First aid kits and other emergency equipment are available across campus with training provided
Psychological crisis intervention services (C4)	2	There are two psychological counselors per 10,000 students, offering psychological services, but coverage is insufficient
Support for students with special health needs (C5)	4	Support is provided to students with special health needs across the five campuses
Cooperation with high-level hospitals (C6)	5	The university's affiliated hospital has agreements with top-tier hospitals to ensure high-level medical services
Timeliness of emergency information release (D1)	3	Emergency information is released quickly, following the prescribed protocol
Actionability of information (D2)	3	Most emergency communications provide clear action guidelines, but about 20–30% lack sufficient guidance
Multi-channel information dissemination (D3)	4	Information is released via multiple official channels to ensure widespread communication
Information error-correction mechanism (D4)	5	Erroneous information is quickly corrected based on clear emergency protocols
Consistency of authoritative information (D5)	4	Information is consistently communicated across platforms, with minor discrepancies
Student emergency liaison roles (D6)	2	Emergency contact roles have been assigned to student leaders, with a ratio of 1:1,000
Academic flexibility and adjustment (E1)	3	The university promptly adjusts teaching plans to minimize academic disruption after emergencies
Coverage of health follow-up services (E2)	4	Health follow-up services are provided to students post-emergency, along with psychological intervention
Provision of basic living conditions (E3)	4	Accommodation and meal support for affected students is provided in a timely and quality-assured manner
Financial assistance and fee reductions (E4)	4	The university provides financial aid and fee waivers to students, with broad coverage for those in need

### 3.2 Weighting results

In the case application, the weights were calculated according to the procedures described in Section 2.2. The subjective weights were derived from the pairwise comparison matrices constructed by 13 experts with experience in university emergency management using the AHP method. The objective weights were calculated using the *CV* method based on the dispersion of indicator scores across ten sampled universities. The basic characteristics of these institutions are presented in Table 6, covering different university types and institutional levels, and reflecting variation in size and regional distribution. The subjective and objective components were then integrated with equal importance (α = 0.5) to generate the comprehensive weights presented in Table 7.

**Table 6:** Basic characteristics of the sampled universities.

No.	University type	Institutional level	Student population ( × 10^4^ persons)	Campus area (km^2^)	Region
U1	Comprehensive	985 / Double first-class	5.5–6.0	4.5–5.0	Central China
U2	Comprehensive	985 / Double first-class	3.5–4.0	3.5–4.0	East China
U3	Comprehensive	985 / Double first-class	6.0–6.5	4.5–5.0	Southwest China
U4	Comprehensive	985 / Double first-class	7.0–7.5	7.0–7.5	Northeast China
U5	Engineering-oriented	211 / Double first-class	4.5–5.0	4.5–5.0	Southwest China
U6	Normal	211 / Double first-class	4.0–4.5	2.0–2.5	Central China
U7	Comprehensive	211 / Double first-class	3.0–3.5	2.5–3.0	South China
U8	Engineering-oriented	Provincial key university	4.0–4.5	2.5–3.0	East China
U9	Engineering-oriented	Provincial key university	3.0–3.5	2.0–2.5	East China
U10	Engineering-oriented	Provincial key university	3.5–4.0	2.5–3.0	Northwest China

**Table 7:** Weight calculation results for criteria and indicators.

Criterion	Subjective weight (criteria layer)	Indicator	Subjective weight (indicator layer)	Global subjective weight	Objective weight (*w*^*obj*^)	Comprehensive weights	$\bar{x_{j}}$	*s*_*j*_	*CV*_*j*_
A	0.2784	A1	0.2604	0.0725	0.0503	0.0614	3.5	1.434	0.410
		A2	0.1313	0.0365	0.0397	0.0381	3.2	1.033	0.323
		A3	0.1089	0.0303	0.0418	0.0361	2.5	0.850	0.340
		A4	0.1462	0.0407	0.0426	0.0416	3.8	1.317	0.346
		A5	0.0824	0.0229	0.0273	0.0251	3.7	0.823	0.223
		A6	0.0779	0.0217	0.0271	0.0244	4.3	0.949	0.221
		A7	0.1862	0.0519	0.0421	0.0470	2.9	0.994	0.343
B	0.1550	B1	0.3121	0.0484	0.0477	0.0481	3.8	1.476	0.388
		B2	0.2264	0.0351	0.0315	0.0333	3.7	0.949	0.256
		B3	0.1011	0.0157	0.0307	0.0232	3.3	0.823	0.249
		B4	0.1768	0.0274	0.0334	0.0304	3.8	1.033	0.272
		B5	0.1614	0.0250	0.0288	0.0269	3.6	0.843	0.234
C	0.2080	C1	0.0961	0.0200	0.0324	0.0262	4.0	1.054	0.264
		C2	0.3015	0.0627	0.0446	0.0536	3.5	1.269	0.363
		C3	0.1687	0.0351	0.0341	0.0346	3.5	0.972	0.278
		C4	0.1485	0.0309	0.0358	0.0333	4.3	1.252	0.291
		C5	0.0664	0.0138	0.0276	0.0207	3.9	0.876	0.225
		C6	0.2124	0.0442	0.0367	0.0404	3.8	1.135	0.299
D	0.1494	D1	0.2792	0.0417	0.0389	0.0403	3.4	1.075	0.316
		D2	0.2212	0.0330	0.0360	0.0345	4.2	1.229	0.293
		D3	0.2073	0.0310	0.0313	0.0311	2.9	0.738	0.254
		D4	0.0728	0.0109	0.0331	0.0220	2.6	0.699	0.269
		D5	0.1370	0.0205	0.0293	0.0249	3.1	0.738	0.238
		D6	0.0753	0.0112	0.0233	0.0172	3.9	0.738	0.189
E	0.1434	E1	0.3954	0.0567	0.0517	0.0542	3.4	1.430	0.421
		E2	0.1528	0.0219	0.0300	0.0260	4.4	1.075	0.244
		E3	0.2487	0.0357	0.0386	0.0371	4.1	1.287	0.314
		E4	0.1740	0.0250	0.0338	0.0294	4.6	1.265	0.275

At the criteria level, as shown in Table 7, Emergency Governance and Operational Effectiveness (A) received the highest comprehensive weight (0.2784), followed by Medical and Health Support Capacity (C) (0.2080). Campus Environment and Facility Safety Readiness (B), Risk Communication and Information Accessibility (D), and Post-Emergency Student Support (E) obtained weights of 0.1550, 0.1494, and 0.1434, respectively. The distribution indicates that governance- and medical-related capacities account for a relatively larger proportion within the overall evaluation structure, whereas the remaining dimensions exhibit comparable weight magnitudes.

At the indicator level, the comprehensive weights demonstrate a differentiated pattern. Specifically, Student-Oriented Emergency Response Procedures (A1, 0.0614), Academic Flexibility and Adjustment (E1, 0.0542), Medical Response Time (C2, 0.0536) and Establishment and Clarity of Emergency Management Authority (A7, 0.0470) obtained relatively higher weights. In contrast, On-Campus Security Personnel Allocation (B3, 0.0232), Information Error-Correction Mechanism (D4, 0.0220), Support for Students with Special Health Needs (C5, 0.0207) and Student Emergency Liaison Roles (D6, 0.0172) received comparatively lower weights. These comprehensive weights were subsequently used both as fixed parameters in FCE and as an importance basis in the priority analysis of observed deficiencies.

### 3.3 Evaluation results

Based on CSU's indicator grades and weights, this study applies a characteristic-function FCE to derive CSU's membership-based resilience scores, as shown in Table 8. The indicator-level results reveal a pronounced disparity between higher- and lower-performing items. Several indicators achieved notably high scores, particularly Comprehensive Evacuation Route Assurance, Academic Flexibility and Adjustment, Medical Response Time, and Provision of Basic Living Conditions, indicating relatively strong performance in physical preparedness, immediate medical response, academic adjustment, and basic post-emergency support. By contrast, Student Emergency Liaison Roles, Decision Documentation and Traceability, and Psychological Crisis Intervention Services recorded markedly lower scores. This contrast suggests that CSU performs considerably better in areas supported by established physical infrastructure and routine operational arrangements than in those requiring student-facing coordination, traceable decision processes, and sustained psychosocial support. Overall, the wide gap between the highest and lowest-scoring indicators points to a clearly uneven resilience profile at the indicator level.

**Table 8:** Criteria-level and indicator-level FCE results.

Indicator	Indicator-layer score	Comprehensive score	Indicator	Indicator-layer score	Comprehensive score
A1	0.897	0.246	C3	0.663	0.138
A2	0.557	0.152	C4	0.319	0.067
A3	0.395	0.108	C5	0.396	0.083
A4	0.608	0.167	C6	0.968	0.202
A5	0.367	0.101	D1	0.711	0.121
A6	0.267	0.073	D2	0.609	0.104
A7	0.687	0.188	D3	0.732	0.124
B1	1.188	0.192	D4	0.646	0.110
B2	0.823	0.133	D5	0.585	0.099
B3	0.716	0.116	D6	0.203	0.034
B4	0.751	0.122	E1	1.109	0.163
B5	0.499	0.081	E2	0.708	0.104
C1	0.627	0.131	E3	1.012	0.148
C2	1.027	0.215	E4	0.801	0.118

**Figure 2:**
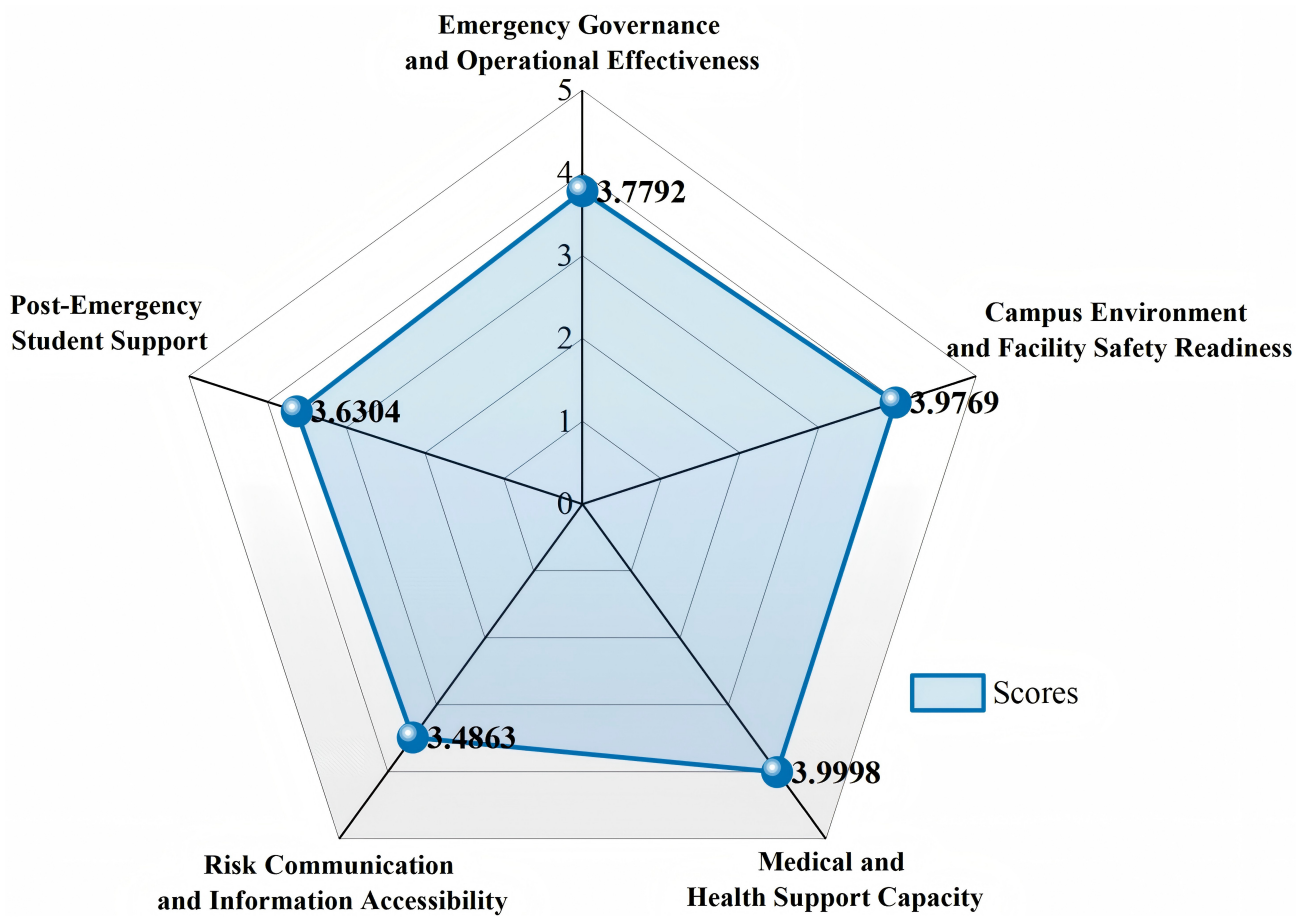
Five-dimension resilience profile of CSU.

Based on the indicator-level membership distributions, a weighted synthesis was performed to obtain the dimension-level membership distributions reported in Table 9; these distributions were then defuzzified using the grade expectation method to derive the dimension scores shown in Figure 2. At the criteria level, Medical and Health Support Capacity achieved the highest score (3.9998), closely followed by Campus Environment and Facility Safety Readiness (3.9769). Emergency Governance and Operational Effectiveness reached 3.7792, followed by Post-Emergency Student Support (3.6304), while Risk Communication and Information Accessibility recorded the lowest score (3.4863).

**Table 9:** The membership degree distribution matrix of five dimensions.

Grade dimension	Very poor	Poor	Average	Good	Excellent
A	0.000	0.000	0.221	0.779	0.000
B	0.000	0.000	0.166	0.691	0.143
C	0.000	0.160	0.000	0.521	0.319
D	0.000	0.101	0.440	0.329	0.129
E	0.000	0.000	0.370	0.630	0.000

By further aggregating the membership degree distribution of five dimensions, the FCE results of CSU are presented in Table 10 (20). The defuzzified comprehensive score of 3.5421 corresponds to Grade 4 (“Good”). The result shows that CSU's UER is generally well developed while still leaving room for further improvement.

**Table 10:** FCE results of CSU.

Very poor	Poor	Average	Good	Excellent	Final score	Final rank
0.0000	0.0483	0.2060	0.5721	0.1078	3.5421	4 (Good)

In conclusion, the evaluation results show a clear structural imbalance in CSU's emergency resilience at both the dimension and indicator levels. Risk Communication and Information Accessibility is the weakest dimension, whereas Campus Environment and Facility Safety Readiness and Medical and Health Support Capacity perform relatively better. To identify which deficiencies should be addressed first, a priority analysis was further conducted.

### 3.4 Priority results

Based on the UER evaluation results of CSU, Equations 11 and 12 were used to analyze the priority of deficiency improvement at both the dimension level and the indicator level. The priority calculation results at the dimension level are presented in Table 11.

**Table 11:** Priority calculation of dimensions.

Dimensions	A	D	C	E	B
Priority	0.340	0.226	0.208	0.196	0.159
Rank	1	2	3	4	5

As shown in Table 11, Emergency Governance and Operational Effectiveness ranked first in priority, followed by Risk Communication and Information Accessibility. Medical and Health Support Capacity and Post-Emergency Student Support occupied intermediate positions, whereas Campus Environment and Facility Safety Readiness ranked lowest. To further identify the specific deficiencies that should be prioritized, indicator-level priority scores were then calculated, as shown in Table 12.

**Table 12:** Priority calculation of indicators.

Indicator	Priority	Rank	Indicator	Priority	Rank
E1	0.108	1	E3	0.037	15
C4	0.100	2	C3	0.035	16
D1	0.081	3	B2	0.033	17
A3	0.072	4	D3	0.031	18
D2	0.069	5	B4	0.030	19
A1	0.061	6	E4	0.029	20
B5	0.054	7	E2	0.026	21
C2	0.054	8	A5	0.025	22
D6	0.052	9	D5	0.025	23
A6	0.049	10	C5	0.021	24
B1	0.048	11	B3	0.000	25
A7	0.047	12	C1	0.000	25
A4	0.042	13	C6	0.000	25
A2	0.038	14	D4	0.000	25

As shown in Table 12, Academic Flexibility and Adjustment (E1) and Psychological Crisis Intervention Services (C4), both with priority scores greater than or equal to 0.10, are the most urgent areas for optimization. Timeliness of Emergency Information Release (D1), Integration of Student Safety and Health Provisions (A3), Actionability of Information (D2), Student-Oriented Emergency Response Procedures (A1), Redundancy of Safety Facilities (B5), Medical Response Time (C2), and Student Emergency Liaison Roles (D6), with scores between 0.05 and 0.10, are also relatively in need of optimization. Indicators with scores below 0.05 but above 0 are relatively less in need of optimization under current conditions, while indicators with scores equal to 0 do not currently require targeted optimization.

Although the dimension-level and indicator-level priority rankings are not fully consistent, this does not indicate a contradiction. Rather, it suggests that CSU's emergency management weaknesses are concentrated in certain domains at the dimensions level, while the most urgent actionable deficiencies are distributed across multiple dimensions at the indicator level. Overall, the evaluation results reveal differentiated performance across dimensions and indicators. To further interpret these patterns and examine their implications for university emergency resilience improvement, the findings are discussed in the following section.

## 4 Discussion

The evaluation results indicate that CSU performs well overall in terms of UER, but still exhibits structural imbalance, as reflected in its relatively strong performance in facility- and treatment-related items, alongside weaker performance in student-facing governance execution, communication usability, psychosocial support, and post-incident continuity management. Further analysis is conducted based on the resilience contribution of indicators. In terms of robustness, the relatively high priority of indicators such as Timeliness of Emergency Information Release, Integration of Student Safety and Health Provisions, actionability of Information, Student-Oriented Emergency Response Procedures, and Medical Response Time suggests that CSU still has room to improve the immediate operability of its core emergency functions. These weaknesses indicate that, although basic emergency resources and institutional arrangements are in place, they have not yet been fully translated into rapid, clear, and student-oriented support in the early stage of emergencies. In terms of recoverability, the prominence of Academic Flexibility and Adjustment and Psychological Crisis Intervention Services suggests that CSU is comparatively weaker in helping students restore academic continuity and psychosocial stability after an incident. This indicates that CSU's resilience remains stronger in immediate response than in post-incident recovery, particularly where sustained academic and mental-health support is required. In terms of redundancy, the relatively high priority of Redundancy of Safety Facilities and Student Emergency Liaison Roles indicates that backup arrangements and alternative student-facing support mechanisms are not yet sufficiently institutionalized. This suggests that CSU's resilience may be constrained when primary facilities, communication channels, or routine support structures are disrupted. Taken together, these patterns suggest that CSU's current resilience is not only uneven across functions, but also marked by a mismatch between relatively stronger physical and medical preparedness and comparatively weaker capacities for sustained recovery and backup-supported student-oriented response. Overall, CSU appears relatively stronger in the “absorbing and responding” stage of resilience, but comparatively weaker in the “coordinating, recovering, and sustaining” stage, especially where emergency management must be translated into practical support for students.

A plausible explanation for this pattern lies in the scale, spatial distribution, and organizational complexity of CSU itself. CSU is a large comprehensive university with more than 62,000 full-time students, 31 secondary colleges, and multiple affiliated high-level hospitals, which provides it with a comparatively strong material and medical base and helps explain its relatively favorable performance in facility readiness and immediate health support. At the same time, emergency management at CSU involves multiple functional actors and systems, including campus security, student affairs, communication mechanisms, and cross-campus support structures, while its psychological support system operates across five sub-centers and serves nearly 3,000 consultation visits per year. This institutional complexity may support broad functional coverage, but it also increases the difficulty of cross-department coordination, unified decision execution, and student-facing information delivery, thereby contributing to the relatively high priority of governance- and communication-related deficiencies. This interpretation is consistent with previous research showing that university crisis communication depends not only on whether information is released, but also on whether communication strategies are perceived as concrete, credible, and actionable by stakeholders (21). In addition, although CSU has an established counseling infrastructure, its psychological support resources mainly function as a broadly distributed service system; under emergency conditions, such a system may be less effective in rapidly absorbing surges in demand for crisis intervention, individualized follow-up, and continuity support across a large multi-campus student population. This helps explain why CSU performs better in physical preparedness and immediate medical response than in psychosocial intervention, academic adjustment, and other recovery-oriented student support functions. The CSU results also offer a more specific conceptual insight into how resilience operates in large universities. The pattern observed here suggests that UER is shaped not only by resource sufficiency, but by the degree of integration across governance, communication, and support systems. CSU benefits from a strong material and medical base, which helps explain its relatively favorable performance in facility readiness and immediate health support. However, as a large comprehensive university with multiple campuses, many academic units, and several student-support systems, CSU also faces higher coordination demands. Under such conditions, the existence of multiple support resources does not automatically produce high resilience. Instead, resilience depends on whether those resources can be synchronized and converted into timely information, coherent action guidance, psychological follow-up, and continuity support for students. This helps explain why governance- and communication-related deficiencies remain highly prioritized despite a relatively strong emergency resource base. It also suggests that, in university settings, the critical distinction may not be between “having” and “not having” emergency resources, but between fragmented capacity and integrated capacity.

This interpretation both confirms and extends the existing literature. Prior research has emphasized organizational structure, emergency planning, communication systems, and psychosocial support as key components of emergency management and resilience in educational settings (1, 3, 8, 13, 22). The present study confirms the relevance of these dimensions, but it also extends current knowledge in several respects. First, it integrates governance, communication, health support, and post-emergency recovery into a unified UER framework explicitly organized around student safety and health. This moves beyond institution-centered assessment by focusing not only on whether emergency arrangements exist, but on whether they function in ways that meaningfully reduce student risk and support student recovery. Second, the findings refine current understanding by showing that student-centered resilience is not equivalent to general institutional preparedness. A university may possess relatively strong physical infrastructure and medical support while still exhibiting weak resilience in communication usability, psychosocial intervention, and academic continuity. Third, the CSU case challenges the implicit assumption that better facilities or stronger immediate response capacity necessarily indicate stronger overall resilience. Here, the most urgent weaknesses are not located in basic physical preparedness, but in the institutional translation of resources into communication, coordination, and continuity-oriented support. This suggests that student-centered assessment can reveal critical vulnerabilities that are less visible in conventional institution-centered approaches. In this regard, the present findings contribute to the broader resilience literature by showing that the effectiveness of university resilience depends heavily on the institutional coupling of immediate response systems with student-oriented recovery and continuity functions.

These findings suggest that CSU's future emergency resilience improvement should focus on how existing institutional resources can better protect student safety and health across the full emergency lifecycle. Given CSU's large student population, multi-college structure, extensive campus space, and strong affiliated-hospital system, the university already has a relatively solid material and medical foundation for emergency response. However, the evaluation and priority results indicate that the more pressing need is to improve how these resources are translated into student-facing protection, support, and continuity functions. In practical terms, CSU should first strengthen cross-department coordination so that security, student affairs, medical services, psychological support, and information release can operate as a more integrated response system, thereby improving the speed and consistency with which students receive protection and assistance during emergencies. Second, because communication-related deficiencies remain highly prioritized, CSU should enhance the timeliness, clarity, and actionability of emergency messages through its existing student-facing platforms and campus-based liaison structures, so that students can more easily understand risks, follow instructions, and reduce avoidable physical and psychological harm. Third, the high priority attached to academic adjustment and psychological crisis intervention indicates that CSU should place greater emphasis on recovery-oriented student support, especially by linking its five-campus psychological support network, extended counseling access, and medical referral resources to more flexible academic arrangements after emergencies. This would not only help reduce the lingering academic and mental-health impacts of emergencies on students, but also strengthen CSU's overall UER by improving its capacity to absorb disruption, sustain essential functions, and support student recovery in a more continuous and student-centered manner (23, 24).

From a practical perspective, the contribution of the evaluation framework proposed in this study lies not merely in assigning an overall resilience score, but in distinguishing between areas of relative strength and those deficiencies that require earlier managerial attention. The combination of weighted evaluation and priority analysis therefore provides a more actionable basis for university emergency planning than a single aggregated index alone. At the same time, the framework should not be treated as a universal template that can be applied without modification. It is likely to be most applicable in institutional settings where emergency management responsibilities are relatively formalized, where student-facing support systems are sufficiently visible, and where documentary or operational evidence is available to support graded assessment. Under such conditions, the framework can help universities align emergency governance with student safety, health, and continuity needs. By contrast, in settings with less formalized governance, weaker support infrastructures, or markedly different hazard profiles, the framework may require contextual adaptation in terms of indicator emphasis, weight structure, and evaluation thresholds. These considerations also shape the interpretation and broader applicability of the present findings.

As an initial empirical application, the study remains subject to several limitations. First, although the framework is explicitly oriented toward student safety and health, the qualitative phase did not fully incorporate the views of a broader range of ordinary students. As a result, the framework may more directly reflect student-facing governance, support, and coordination concerns than the full diversity of student experiences and vulnerabilities, which may limit its direct applicability in institutional contexts where ordinary students' everyday perceptions and informal coping needs are more salient. Second, the framework was specifically designed from the perspective of student safety and health and therefore does not encompass every aspect of institutional emergency management. Third, although the study constructs a relatively comprehensive framework for evaluating UER, it focuses primarily on resilience-related capacities and does not examine in depth how these capacities are translated into actual emergency outcomes. Fourth, the empirical validation is based on a single-case application at CSU, so the framework may be analytically transferable, but the specific performance pattern and priority ordering reported here should be interpreted as context-sensitive rather than universally generalizable. Finally, the present assessment remains static and capacity-oriented, identifying resilience-related strengths and weaknesses at a particular point in time without capturing how UER evolves across different emergency stages, hazard types, and institutional contexts. Future research could therefore strengthen the framework by incorporating a broader range of student perspectives, testing it across different university types and governance settings, further examining the relationship between UER and actual emergency outcomes, and applying longitudinal or comparative designs. In this way, the proposed framework may be further refined from an exploratory student-centered assessment model into a more broadly applicable tool for evidence-based university emergency resilience planning.

## 5 Conclusion

This study developed and applied a student-centered framework for evaluating university emergency resilience and prioritizing improvement actions. Using CSU as the empirical case, the results showed that CSU achieved a generally good level of UER (3.5421, Grade 4), but with a clear structural imbalance: Medical and Health Support Capacity and Campus Environment and Facility Safety Readiness performed relatively well, whereas Risk Communication and Information Accessibility remained the weakest dimension. The priority analysis further indicated that Academic Flexibility and Adjustment, Psychological Crisis Intervention Services, and several governance- and communication-related capacities should be addressed first. By integrating student safety, health, communication, and post-emergency recovery into a unified assessment framework and linking weighted evaluation with priority analysis, this study extends institution-centered approaches to UER assessment and provides a more actionable basis for universities to identify and prioritize resilience improvement efforts.

## Data Availability

The original contributions presented in the study are included in the article/Supplementary material, further inquiries can be directed to the corresponding author.
