# Supplementary material for: Evaluating university emergency resilience with a focus on student safety and health
Source: Front Public Health. 2026 May 14;14:1830542. doi: 10.3389/fpubh.2026.1830542 (PMC13216232; doi:10.3389/fpubh.2026.1830542)
Supplement: Supplementary file 1 [file Table_1.docx]

**Questionnaire for AHP Weight Determination of University Emergency Resilience Indicators**

Research note: This questionnaire is intended for academic use only. It aims to determine the relative importance of university emergency resilience criteria and indicators from a student safety and health perspective using the Analytic Hierarchy Process (AHP). All information will be kept confidential and used solely for research purposes.

# 1. Research Objective

The questionnaire is designed to support the construction of a University Emergency Resilience evaluation framework. Experts are invited to compare the relative importance of criteria and indicators with respect to the overall goal of evaluating university emergency resilience focused on student safety and health.

The framework contains five criteria: Emergency Governance and Operational Effectiveness; Campus Environment and Facility Safety Readiness; Medical and Health Support Capacity; Risk Communication and Information Accessibility; and Post-Emergency Student Support.

# 2. Instructions for Pairwise Judgment

Please compare the relative importance of two elements with respect to the overall evaluation goal. When the row element is more important than the column element, enter 1, 3, 5, 7, 9, or an intermediate value. When the column element is more important, use the reciprocal value. You may fill only the upper triangular cells. The diagonal entries are fixed at 1, and the lower triangular cells will be completed by the reciprocal rule.

| **Scale** | **Meaning** |
| --- | --- |
| 1 | Equal importance |
| 3 | Moderate importance of one element over another |
| 5 | Strong importance |
| 7 | Very strong importance |
| 9 | Extreme importance |
| 2, 4, 6, 8 | Intermediate values between adjacent judgments |
| Reciprocal | If the column element is more important than the row element, use the reciprocal value |

# 3. Expert Information

| **Item** | **Response** |
| --- | --- |
| Expert code / Name |  |
| Institution |  |
| Position / Title |  |
| Years of relevant experience |  |
| Primary field of expertise |  |
| Familiarity with university emergency management practice | □ Very familiar □ Familiar □ Basically familiar □ Slightly familiar □ Not familiar |

# 4. Criteria Layer Pairwise Comparison

Overall evaluation goal: Evaluating University Emergency Resilience from a student safety and health perspective.

Criteria definitions:

- A. Emergency Governance and Operational Effectiveness
- B. Campus Environment and Facility Safety Readiness
- C. Medical and Health Support Capacity
- D. Risk Communication and Information Accessibility
- E. Post-Emergency Student Support

## Table 1. Criteria Layer Judgment Matrix

|  | **A** | **B** | **C** | **D** | **E** |
| --- | --- | --- | --- | --- | --- |
| A | 1 |  |  |  |  |
| B |  | 1 |  |  |  |
| C |  |  | 1 |  |  |
| D |  |  |  | 1 |  |
| E |  |  |  |  | 1 |

# 5. Indicator Layer Pairwise Comparison under Criterion A

**Criterion A. Emergency Governance and Operational Effectiveness**

- A1. Student-oriented emergency response procedures
- A2. Emergency communication channels
- A3. Integration of student safety and health provisions
- A4. Student participation in emergency training and drills
- A5. Post-incident review and improvement mechanism
- A6. Decision documentation and traceability
- A7. Establishment and clarity of emergency management authority

## Table 2. Judgment Matrix for A1, A2, A3, A4, A5, A6, A7

|  | **A1** | **A2** | **A3** | **A4** | **A5** | **A6** | **A7** |
| --- | --- | --- | --- | --- | --- | --- | --- |
| A1 | 1 |  |  |  |  |  |  |
| A2 |  | 1 |  |  |  |  |  |
| A3 |  |  | 1 |  |  |  |  |
| A4 |  |  |  | 1 |  |  |  |
| A5 |  |  |  |  | 1 |  |  |
| A6 |  |  |  |  |  | 1 |  |
| A7 |  |  |  |  |  |  | 1 |

# 6. Indicator Layer Pairwise Comparison under Criterion B

**Criterion B. Campus Environment and Facility Safety Readiness**

- B1. Comprehensive evacuation route assurance
- B2. Campus safety facilities
- B3. On-campus security personnel allocation
- B4. Continuity of essential public services
- B5. Redundancy of safety facilities

## Table 3. Judgment Matrix for B1, B2, B3, B4, B5

|  | B1 | B2 | B3 | B4 | B5 |
| --- | --- | --- | --- | --- | --- |
| B1 | 1 |  |  |  |  |
| B2 |  | 1 |  |  |  |
| B3 |  |  | 1 |  |  |
| B4 |  |  |  | 1 |  |
| B5 |  |  |  |  | 1 |

# 7. Indicator Layer Pairwise Comparison under Criterion C

**Criterion C. Medical and Health Support Capacity**

- C1. Campus hospital capacity
- C2. Medical response time
- C3. Emergency medical equipment coverage
- C4. Psychological crisis intervention services
- C5. Support for students with special health needs
- C6. Cooperation with high-level hospitals

## Table 4. Judgment Matrix for C1, C2, C3, C4, C5, C6

|  | **C1** | **C2** | **C3** | **C4** | **C5** | **C6** |
| --- | --- | --- | --- | --- | --- | --- |
| C1 | 1 |  |  |  |  |  |
| C2 |  | 1 |  |  |  |  |
| C3 |  |  | 1 |  |  |  |
| C4 |  |  |  | 1 |  |  |
| C5 |  |  |  |  | 1 |  |
| C6 |  |  |  |  |  | 1 |

# 8. Indicator Layer Pairwise Comparison under Criterion D

**Criterion D. Risk Communication and Information Accessibility**

- D1. Timeliness of emergency information release
- D2. Actionability of information
- D3. Multi-channel information dissemination
- D4. Information error-correction mechanism
- D5. Consistency of authoritative information
- D6. Student emergency liaison roles

## Table 5. Judgment Matrix for D1, D2, D3, D4, D5, D6

|  | **D1** | **D2** | **D3** | **D4** | **D5** | **D6** |
| --- | --- | --- | --- | --- | --- | --- |
| D1 | 1 |  |  |  |  |  |
| D2 |  | 1 |  |  |  |  |
| D3 |  |  | 1 |  |  |  |
| D4 |  |  |  | 1 |  |  |
| D5 |  |  |  |  | 1 |  |
| D6 |  |  |  |  |  | 1 |

# 9. Indicator Layer Pairwise Comparison under Criterion E

**Criterion E. Post-Emergency Student Support**

- E1. Academic flexibility and adjustment
- E2. Coverage of health follow-up services
- E3. Provision of basic living conditions
- E4. Financial assistance and fee reductions

## Table 6. Judgment Matrix for E1, E2, E3, E4

|  | **E1** | **E2** | **E3** | **E4** |
| --- | --- | --- | --- | --- |
| E1 | 1 |  |  |  |
| E2 |  | 1 |  |  |
| E3 |  |  | 1 |  |
| E4 |  |  |  | 1 |

# 10. Open-Ended Questions

1. Are there any indicators that you consider unclear, overlapping, or difficult to judge? Please explain.

________________________________________________________________________________________

________________________________________________________________________________________

1. Are there any important indicators related to student safety and health in university emergencies that are missing from the current framework? Please explain.

________________________________________________________________________________________

________________________________________________________________________________________

**Questionnaire for indicator consistency test of University Emergency Resilience**

# 1. Research Objective

This questionnaire is designed to validate the indicator system developed for evaluating university emergency resilience (UER) from the perspective of student safety and health. Experts are invited to assess the substantive importance and practical appropriateness of each proposed indicator.

**2. Response instruction**

Please rate each indicator on a 1–9 scale according to its importance for evaluating UER. Higher scores indicate greater importance, clearer relevance to the evaluation objective, and stronger necessity for inclusion in the final framework.

| **Score** | **Interpretation** |
| --- | --- |
| 1 | Not important / should not be prioritized in the evaluation framework |
| 3 | Slightly important |
| 5 | Moderately important |
| 7 | Very important |
| 9 | Extremely important / essential indicator |
| 2, 4, 6, 8 | Intermediate values between adjacent judgments |

**3. Expert Information**

| **Item** | **Response** |
| --- | --- |
| Expert code / Name |  |
| Institution |  |
| Position / Title |  |
| Years of relevant experience |  |
| Primary field of expertise |  |
| Familiarity with university emergency management practice | □ Very familiar □ Familiar □ Basically familiar □ Slightly familiar □ Not familiar |

**4. Indicator Rating Form**

Please assign one score from 1 to 9 to each indicator based on its importance for evaluating UER from the perspective of student safety and health. You may add brief comments where necessary.

**A. Emergency Governance and Operational Effectiveness**

| **Code** | **Indicator** | **Operational description** | **Score (1–9)** |
| --- | --- | --- | --- |
| **A1** | Student-Oriented Emergency Response Procedures | Whether clear, documented, and operational emergency response procedures for students have been established. |  |
| **A2** | Emergency Communication Channels | Number and accessibility of official emergency communication channels available to students during emergencies. |  |
| **A3** | Integration of Student Safety and Health Provisions | Extent to which student safety and health requirements are embedded in university emergency regulations and plans. |  |
| **A4** | Student Participation in Emergency Training and Drills | Frequency and coverage of student participation in emergency education, training, and drills. |  |
| **A5** | Post-Incident Review and Improvement Mechanism | Whether formal review, feedback, and corrective improvement mechanisms are institutionalized after incidents. |  |
| **A6** | Decision Documentation and Traceability | Completeness, standardization, and traceability of emergency decision records and response logs. |  |
| **A7** | Establishment and Clarity of Emergency Management Authority | Clarity of emergency management responsibilities, command relationships, and interdepartmental authority arrangements. |  |

**B. Campus Environment and Facility Safety Readiness**

| **Code** | **Indicator** | **Operational description** | **Score (1–9)** |
| --- | --- | --- | --- |
| **B1** | Comprehensive Evacuation Route Assurance | Whether evacuation routes are clearly identified, unobstructed, and routinely inspected. |  |
| **B2** | Campus Safety Facilities | Coverage, functionality, and maintenance of core campus safety facilities such as alarms, firefighting systems, and emergency lighting. |  |
| **B3** | On-Campus Security Personnel Allocation | Adequacy of campus security staffing for emergency response support and order maintenance. |  |
| **B4** | Continuity of Essential Public Services | Ability to maintain or rapidly restore essential services such as electricity, water, and basic operational support. |  |
| **B5** | Redundancy of Safety Facilities | Availability of backup or redundant safety facilities, equipment, and supporting infrastructure. |  |

**C. Medical and Health Support Capacity**

| **Code** | **Indicator** | **Operational description** | **Score (1–9)** |
| --- | --- | --- | --- |
| **C1** | Campus Hospital Capacity | Adequacy of campus medical facilities, staffing, and treatment capacity for emergency support. |  |
| **C2** | Medical Response Time | Timeliness with which medical personnel or emergency health support can reach affected students. |  |
| **C3** | Emergency Medical Equipment Coverage | Coverage and availability of AEDs, first-aid kits, stretchers, and related emergency medical equipment. |  |
| **C4** | Psychological Crisis Intervention Services | Availability and accessibility of psychological crisis intervention and mental health support during and after emergencies. |  |
| **C5** | Support for Students with Special Health Needs | Extent to which emergency arrangements consider and support students with disabilities, chronic illness, or other special health needs. |  |
| **C6** | Cooperation with High-Level Hospitals | Effectiveness of referral pathways and cooperative arrangements with external high-level hospitals. |  |

**D. Risk Communication and Information Accessibility**

| **Code** | **Indicator** | **Operational description** | **Score (1–9)** |
| --- | --- | --- | --- |
| **D1** | Timeliness of Emergency Information Release | Speed with which official emergency information is released to students after an incident emerges. |  |
| **D2** | Actionability of Information | Whether communication content clearly tells students what actions they should take. |  |
| **D3** | Multi-Channel Information Dissemination | Diversity and coordination of channels used to disseminate emergency information to students. |  |
| **D4** | Information Error-Correction Mechanism | Ability of the university to identify, correct, and contain inaccurate or misleading information in a timely manner. |  |
| **D5** | Consistency of Authoritative Information | Consistency of official emergency information across departments and communication platforms. |  |
| **D6** | Student Emergency Liaison Roles | Whether student liaison or coordination roles are established to support information transmission and response implementation. |  |

**E. Post-Emergency Student Support**

| **Code** | **Indicator** | **Operational description** | **Score (1–9)** |
| --- | --- | --- | --- |
| **E1** | Academic Flexibility and Adjustment | Capacity to adjust teaching schedules, attendance, assessments, and academic arrangements after emergencies. |  |
| **E2** | Coverage of Health Follow-Up Services | Availability of continued health, rehabilitation, or follow-up services after the immediate emergency stage. |  |
| **E3** | Provision of Basic Living Conditions | Ability to provide temporary accommodation, meals, and basic living support for affected students. |  |
| **E4** | Financial Assistance and Fee Reductions | Availability of financial aid, subsidies, or fee-reduction measures for students affected by emergencies. |  |

**5. Open-Ended Feedback**

1. Are any indicators unclear, overlapping, or difficult to judge? Please specify.

________________________________________________________________________________________

________________________________________________________________________________________

2. Are there any important indicators related to student safety and health in university emergencies that are missing from the current framework?

________________________________________________________________________________________

________________________________________________________________________________________
